# Recognizing vaccine wastage in Georgia

**DOI:** 10.1002/puh2.46

**Published:** 2022-12-12

**Authors:** Giorgi Aladashvili, Anano Nebieridze, Giorgi Pkhakadze, Ilia Nadareishvili

**Affiliations:** ^1^ School of Public Health David Tvildiani Medical University Tbilisi Georgia

**Keywords:** Georgia, hesitancy, policies, Tbilisi, vaccine wastage

## Abstract

The COVID‐19 vaccination program in the country of Georgia began on March 15, 2021, and reached its peak in the summer of 2021. Throughout the process, individuals had access to over 5.3 million doses of COVID‐19 vaccines which were acquired from various sources as reported by the National Center for Disease Control and Public Health (NCDC). Factors such as widespread vaccine hesitancy and low demand led to reduced vaccine uptake, low vaccination rates, and vaccine wastage. As of August 2022, a total of 2,922,000 doses have been administered with less than 35% of the Georgian population, or 1,276,000 people, fully vaccinated with at least two doses. Over 400,000 doses have expired at NCDC's storage facilities due to low demand. Many more doses have been wasted at administration points, and there is a risk of wasting more in the future. The key reasons for wastage are widespread public hesitancy toward the newly developed vaccines, inconsistent policies and communication from government officials, rampant disinformation, and ambiguity from influential bodies in Georgian society. Despite certain limitations, logistics is not among the leading causes of wastage, as Georgia was able to develop a strong cold‐chain and vaccine administration infrastructure through years of international cooperation that allowed for efficient management of the COVID‐19 vaccination process. Lastly, in addition to establishing a robust reporting system and ensuring transparency of vaccine wastage data, relevant studies based on original data are required to better understand the problem. Conducting studies on health literacy in the country as a baseline for long‐term interventions, as well as research that will increasingly evidence‐inform further pandemic response are being recommended.

## BACKGROUND

Georgia is an Eastern European country of 3.7 million, which became a member state of the United Nations in 1992. Since then, the healthcare system of Georgia has become progressively decentralized. Currently, over 90% of healthcare facilities are operated as private “for‐profit” institutions. A chronic weakness of this increasingly privatized sector has been primary health care (PHC). This “weakness” became especially apparent with the advent of the COVID‐19 pandemic, as the PHC sector in Georgia endeavored to build support for and confidence in COVID‐19 vaccines and distribute them equitably.

Large‐scale vaccination campaigns, which are often viewed as the principal means of addressing the COVID‐19 pandemic, have faced significant global challenges, including in the European region [1]. Georgia, which by September 2022 had reported over 1,720,000 cases and 16,900 deaths attributed to COVID‐19, started vaccinations on March 15, 2021. The country's COVID‐19 vaccination plan, approved in early 2021, aimed to vaccinate 60% of the adult population, or 1.7 million people by the end of 2021 [2]. This plan faced several obstacles, chiefly vaccine hesitancy. Polling data from February 2021, indicated a high baseline rate of vaccine hesitancy in the general population [3].

The vaccine rollout began on March 15, 2021, initially prioritizing healthcare workers, but only a small fraction of them (∼10%) scheduled their vaccinations. Soon after, as the scale of hesitancy towards the new vaccine exceeded initial expectations, the national COVID‐19 vaccine rollout plan was effectively abandoned. Instead, information campaigns regarding COVID‐19 vaccine safety and efficacy intensified from spring 2021, and individuals were encouraged to get vaccinated through conventional mass media, as well as online and social media platforms [4].

As the general population became more receptive and larger quantities of both donated and procured vaccines reached the country, a mass vaccination process began in the summer of 2021. During the 2021 vaccination process, according to the National Center for Disease Control and Public Health (NCDC), individuals could choose from Pfizer‐BioNTech, AstraZeneca, Sinopharm, and Sinovac (CoronaVac) vaccines and had access to over 5 million doses imported throughout the year [4].

Access to the vaccination process did not extend to communities within the 20% of Georgian territories occupied by Russia due to very limited ability of individuals to cross into Georgian government‐controlled territories, and refusal of local de facto governments to receive vaccine aid from Tbilisi. This disadvantaged individuals in these communities in their vaccination choice and opportunities, with access to only the Russian‐made Sputnik‐V.

Public demand for COVID‐19 vaccines wavered during the fall of 2021, and the Georgian government introduced monetary incentives and further educational interventions to reinvigorate the vaccination process. Concurrently, the government avoided potentially unpopular vaccination mandates, and only issued directives urging the vaccination of public service, hospitality, and healthcare industry workers. Government interventions were complemented by pro‐vaccine efforts by civic society, international organizations (e.g. WHO, UNICEF, USAID), and healthcare workers.

The Georgian government remained inconsistent with its COVID‐19 public health policies, which detracted from its prior efforts. The introduction of COVID‐19 “green passports” on December 1, 2021, intended to identify fully vaccinated, recovered, or laboratory‐proven SARS‐CoV‐2 negative individuals, was the most notable example of this inconsistency [5]. This system functioned for a mere 2 months and was deactivated on February 1, 2022, as it was claimed to be obsolete and lacking efficacy. This added to existing controversy and confusion among the general public. Furthermore, statements made by high‐ranking officials could have discouraged people from getting vaccinated.

By early 2022, the vaccination process had stalled as demand became extremely minimal. NCDC reported that, as of August 21, 2022, 2,922,000 doses of COVID‐19 vaccines had been administered, but only 1,276,000 people, or less than 35% of the Georgian population, had been “fully” vaccinated with at least two doses.

## VACCINE WASTAGE

Vaccine wastage ‐ a common reality of any vaccination program, received significant attention during the COVID‐19 pandemic. Although some wastage was to be expected, unnecessary and preventable wastage has emerged as reasonable concern. Some countries reported wastage rates as high as 1/3rd and over 1 billion doses were estimated to be wasted globally as of July 2022 [6, 7]. As COVID‐19 vaccines represent a global public good, their wastage irrespective of location or source (e.g., donated or procured) contributes to global economic loss and inequity [8].

The high vaccine wastage in high and middle‐income countries and the lack of access to COVID‐19 vaccines by millions in lower‐income countries have created a global ethical dilemma in equitable vaccine allocation strategies. In the case of Georgia, vaccine wastage poses a significant risk to the majority of Georgian residents yet to be vaccinated and represents a potential economic loss for the Georgian government.

There is limited public data available on vaccine wastage in Georgia. Information is derived from online news reports and published interviews with government officials. The extrapolation of data from these sources is unrealistic as they vary significantly. This led the authors to contact the NCDC to inquire about the official information about the number of vaccines acquired and estimates of their wastage. Table 1 shows how Georgia had acquired 5,334,060 doses of COVID‐19 vaccines, from which 408,801 doses were wasted due to expiration as of August 30, 2022.

**TABLE 1:** COVID‐19 vaccines received by Georgia by sources and wasted doses

Vaccine	Source	Received doses	Wasted doses	Administered doses *(according to vaccines.ncdc.ge)*	Doses stored at possible risk of wastage^a^
Pfizer	Procured by state	1,279,980	0	1,710,428	278,170
	Donated	1,104,480	395,862		
Sinopharm	Procured by state	1,600,000	0	839,136	860,864
	Donated	100,000	0		
Sinovac	Procured by state	1,000,000	1,619	244,580	855,420
	Donated	100,000	0		
AstraZeneca	Procured by state	129,600	0	122,021	0^b^
	Donated	20,000	11,320		

^a^ These numbers reflect our approximation taking into account the numbers provided by the NCDC on received doses and doses expired (wasted) at their storage facility only and do not account for possible doses wasted at other logistic levels.

^b^ AstraZeneca has been out of use in Georgia since fall of 2021.

Surprisingly, despite Pfizer‐BioNTech being highly sought as a vaccine, it still accounted for 97% or approximately 400,000 doses of the vaccines that were wasted due to expiration. Additional doses must have been wasted at provider points, most likely due to open vial wastage. For example, Georgia had received 149,600 doses of the AstraZeneca vaccine, of which 122,021 were administered and 11,320 were wasted at the storage level. Thus, it can be assumed that the remaining 16,000 doses were wasted at provider points, as AstraZeneca has been out of use in Georgia since late 2021.

Similar estimates can be made for the 2.8 million doses of Sinopharm and Sinovac vaccines acquired by Georgia. Out of these, 1.6 million doses with an expiration date in June‐July of 2023 were stored as of March 2022. While an additional 1,083,716 doses were administered by the end of August 2022, and just 1,619 doses had expired. It can be reasonably assumed that the remaining unaccounted doses were wasted as a result of open vial wastage or other factors. Despite less stringent storage requirements and longer shelf lives, stored doses of Sinopharm and Sinovac vaccines are at risk of expiration as public demand for additional vaccinations remains very low.

## WHAT COULD BE THE KEY REASON FOR WASTAGE?

There are no research studies providing specific reasons for vaccine wastage in Georgia. It is surmised that the underlying cause lies in widespread public hesitancy toward the newly developed vaccines, inconsistent communication from government officials, widespread disinformation, anti‐vaccination movements, and apparent hesitancy from a significant portion of Georgian health workers. This low‐demand environment promotes underutilization of acquired vaccine stores, leading to expiration and multi‐dose vials wastage.

Lack of a clear stance from the Georgian Orthodox Church, arguably one of the most trusted and influential organization in Georgia, also contributed to vaccine hesitancy and wastage. The Georgian Orthodox Church was supportive of initial measures such as lockdowns and social distancing, but took a neutral stance with regard to COVID‐19 vaccines, neither encouraging nor prohibiting inoculation. Similar to the Church of Antioch, it viewed the advice on vaccination as the responsibility of medical specialists rather than the church's. Similar stances were not taken by many individual priests and bishops who expressed their strongly negative attitude toward COVID‐19 vaccines, while other Orthodox primates such as the Ecumenical Patriarch called on people to get vaccinated to protect their own and others’ lives. Overall, the environment observed in Georgia in this regard strongly resembled that of other Eastern Orthodox societies in countries like Romania, Greece, or Russia [9].

In addition to the above‐mentioned causes, hesitancy and mistrust towards the vaccination process can also be traced back to the growing lack of confidence towards physicians in Georgia, as well as systemic challenges in the medical practice such as limited communication, patient counseling, and preventive care provided to Georgian citizens [10]. The fundamental reason for these issues most likely lies in the insufficient continuing medical education and professional development of a majority of medical professionals, and growing challenges in both undergraduate and postgraduate medical education in Georgia [11].

Logistics (transportation, storage, etc.) is not seen to have contributed significantly to vaccine wastage. This is attributed to years of international cooperation and partnership with organizations such as the US Centers for Disease Control and Prevention (CDC Atlanta), UNICEF, and the World Health Organization, Georgia has developed a strong cold‐chain and vaccination administration infrastructure that allowed for efficient logistical management of the process. Despite this progress, there are still regulatory financial and infrastructural setbacks, personnel shortages in low‐demand areas, and healthcare disparities across the urban‐rural divide which represent underlying challenges to immunization programs [12, 13].

## CONCLUSION AND RECOMMENDATIONS

The vaccine wastage rate in Georgia has been relatively moderate relative to the population size. Continued vaccine hesitancy and low demand for excess vaccines currently in store increases the risk of additional vaccine wastage. Several actions had been recommended in order to reduce COVID‐19 vaccine wastage [6]. These include establishment of transparent reporting systems that will allow identification of chief determinants of vaccine wastage and ways in which wastage can be minimized; as well as improved logistical management to prevent open vial wastage. Studies on health literacy should be conducted, as a baseline for long‐term interventions aimed at addressing the highly prevalent hesitancy to vaccines, improving health literacy, and building a healthier society in general. Academic and professional efforts need to be focused on research to evidence‐inform pandemic response in areas such as vaccination planning, vaccine procurement and administration strategies, which could help avoid issues such as excess or deficits of vaccines. Georgia needs systemic, multiscale and multilevel reforms in health, spanning education, governance, healthcare provision and other domains.

## AUTHOR CONTRIBUTIONS

Ilia Nadareishvili and Giorgi Aladashvili conceived the idea of the present manuscript; Ilia Nadareishvili wrote the first draft and supervised the scientific and manuscript writing processes; Giorgi Aladashvili reviewed and edited the first draft; All authors contributed to data acquisition, editing the manuscript, reviewing and approving the final manuscript.

## CONFLICT OF INTEREST

The authors report no conflicts of interest.

## ETHICS STATEMENT

This is not an original study but a commentary paper which doesn't use any originally collected data and didn't require Ethical Committee review or approval.

## Data Availability

This is a commentary paper and we don't have original data to share.
